# A molecular census to elucidate the demixing mechanism of membraneless organelles

**DOI:** 10.1186/s13059-025-03806-0

**Published:** 2025-10-09

**Authors:** Cheryn Ali, Fernando Muzzopappa, Fabian Erdel

**Affiliations:** https://ror.org/004raaa70 grid.508721.9 0000 0001 2353 1689 MCD, Center for Integrative Biology (CBI), University of Toulouse, CNRS, Toulouse, France

**Keywords:** Membraneless organelles, Condensates, Phase separation, Demixing mechanisms, Biology by the numbers, Debye length, Percolation, Network fluid

## Abstract

**Background:**

Cells contain membraneless organelles that have been proposed to form via phase separation involving dense networks of multivalent intermolecular interactions. As it is notoriously difficult to experimentally distinguish punctate structures formed by phase separation from those formed by other mechanisms, this issue is controversial. To complement experimental assays, we present a computational by-the-numbers approach to phase separation. We mine publicly available datasets to perform a molecular census of prominent subnuclear organelles in mouse embryonic stem cells: nucleoli, transcriptional condensates, heterochromatin foci, and Polycomb bodies. We estimate copy numbers and intermolecular distances and compare the latter to the Debye length, which is the characteristic distance over which intermolecular interactions typically occur.

**Results:**

We find that none of the organelles studied here contain any protein species that shows intermolecular distances below the estimated Debye length if molecules in the organelles are randomly distributed, which disfavors the classical one-component phase separation scenario. Considering multiple species based on databases of phase-separating proteins, we find that nucleoli and transcriptional condensates are compatible with multi-component phase separation driven by proteins and RNAs, while heterochromatin foci and Polycomb bodies are better explained by a model in which proteins bind to chromatin without phase-separating via dense multivalent interaction networks. We also provide an interactive tool that allows testing of alternative multi-component scenarios.

**Conclusion:**

We introduce a computational by-the-numbers approach to benchmark different demixing models that may explain the assembly of membraneless organelles. Our results suggest that cells use different mechanisms to form subnuclear organelles with different biophysical properties.

**Supplementary Information:**

The online version contains supplementary material available at 10.1186/s13059-025-03806-0.

## Background

**Fig. 1:**
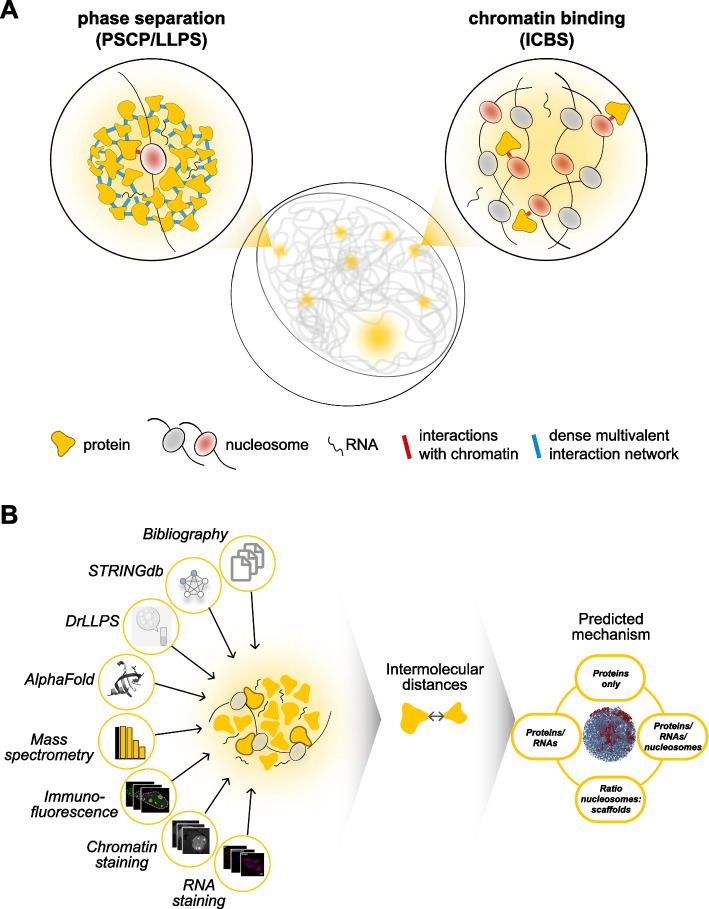
Computational workflow to elucidate the molecular content and demixing mechanism of subnuclear organelles. **A** Molecular mechanisms that can drive demixing of molecules in the cell nucleus. *PSCP* phase separation coupled to percolation, *LLPS* liquid–liquid phase separation, *ICBS* interaction with clustered binding sites. Demixing might go along with conformational changes of the nucleosome chain and polymer–polymer phase separation, which we did not consider here for simplicity. **B** Strategy to perform a molecular census of subnuclear organelles based on public resources, which is used to determine intermolecular distances to elucidate the underlying demixing mechanism

Cells separate different biological processes from each other using membraneless subcompartments [1]. Many of these have been proposed to form via a phase separation mechanism that explains demixing of molecules from a homogenous solution via multivalent interactions (Fig. 1A, left). In this scenario, each molecule interacts simultaneously with multiple neighboring molecules, creating a dense network of interactions [2]. These interactions can be homotypic self-interactions among molecules of the same type, or heterotypic interactions among molecules of different types [2–4]. As intermolecular interactions can only act over a limited distance at the nanometer scale [5–7], which corresponds to the so-called Debye length [8], intermolecular distances among phase-separating scaffold molecules are in this scenario necessarily quite small. The underlying demixing mechanism has been referred to as liquid–liquid phase separation (LLPS) [9] and more recently as phase separation coupled to percolation (PSCP), highlighting the role of dense percolating interaction networks in the condensates [10]. Besides phase separation, there are other mechanisms to explain the demixing of molecules from solution, especially in the cell nucleus that contains large chromosomes [11]. In particular, site-specific interactions of molecules with clustered binding sites (ICBS) on chromosomes can also drive their demixing from solution (Fig. 1A, right). ICBS does not require any multivalent interactions but can occur via low-valency interactions between RNA/protein molecules and chromatin [12, 13], which are often mediated by “reader” domains that recognize DNA sequence motifs or posttranslational histone modifications [14]. Intermolecular distances among the chromatin-bound RNA/protein molecules in such a structure can be much larger than the Debye length, as molecules are held in place by the chain of nucleosomes they are bound to, and a percolating network of multivalent interactions is not required. RNAs/proteins in such structures are often referred to as clients, with the chain of nucleosomes representing the only scaffold [4]. Both types of demixing, PSCP/LLPS and ICBS, might involve conformational changes of the nucleosome chain and polymer–polymer phase separation [11, 15, 16], which we do not consider here for simplicity. Membraneless organelles formed by PSCP/LLPS have distinct properties that are caused by their dense multivalent interaction networks. The latter can create a specific physicochemical microenvironment, going along with altered solution properties and the selective partitioning of molecules in condensates [1, 17, 18]. Multivalent interactions linked to PSCP/LLPS can also create an interface that attenuates molecular exchange between the condensate and its surroundings and that can generate mechanical forces [12, 19, 20]. Dense interaction networks and their consequences have recently been proposed to be druggable, providing a basis for the development of new classes of condensate-modifying drugs [21].

It is currently debated how to experimentally distinguish between the above-mentioned mechanisms in cells [22–24]. Both membraneless organelles formed by phase separation (PSCP/LLPS) and those formed by ICBS can appear as “puncta” under the microscope, exhibit fast molecular turnover, and be sensitive or insensitive to the aliphatic alcohol 1,6-hexanediol [12]. To address this challenge, we introduce here a computational by-the-numbers approach [25], which uses public datasets in conjunction with simple principles from molecular biophysics to shed light on the mechanism underlying membraneless organelles (Fig. 1B). We estimated the copy numbers of candidate scaffold molecules in four types of membraneless organelles of mouse embryonic stem cell (mESC) lines, and we used them to estimate intermolecular distances. Based on the simple principle from physics that molecules can only interact if they are spatially close enough to each other, we compared the different demixing mechanisms. Our approach integrates complementary data types obtained in unperturbed cells, and largely avoids model-specific assumptions about the specific behavior of the respective molecules.

We focused on four prominent subnuclear organelles: nucleoli, transcriptional condensates, heterochromatin foci, and Polycomb bodies. Based on a set of assumptions that reflects our current knowledge about these organelles, we found that transcriptionally active nucleoli and transcriptional condensates are compatible with multicomponent PSCP/LLPS-type phase separation, in which all RNAs and candidate scaffold proteins in the organelles are close enough to multivalently interact with each other. Nucleoli also contain an excess of candidate scaffold proteins over nucleosomes, disfavoring ICBS as the abundance of binding sites on chromatin is limited. Transcriptionally silent heterochromatin foci and Polycomb bodies feature quite large intermolecular distances, even if all known candidate scaffolds are considered together, which is in line with ICBS. Heterochromatin foci and Polycomb bodies contain an excess of nucleosomes that can serve as binding sites. When individual candidate scaffold proteins in any of the four organelles are considered in isolation, none of them exhibits intermolecular distances that are compatible with PSCP/LLPS, indicating that the homotypic self-interactions routinely assayed with single protein species in vitro are not prevalent in the respective cellular organelles. RNAs may be key ingredients that can increase the network density in transcriptionally active organelles. Our results suggest that cells use different mechanisms to assemble subnuclear organelles with different activities.

## Results

### A computational strategy to perform a molecular census of subnuclear organelles

We performed a molecular census of subnuclear membraneless organelles in silico, focusing on molecules that have been proposed in the literature to phase-separate (Fig. 1B). We first selected a prominent marker protein for each subnuclear organelle, which is enriched in the organelle and has been proposed to drive phase separation. We subsequently used the STRING database [26] to retrieve a list of known interactors, using a relatively low stringency to include transient interactors (Additional file 1: Fig. S1). We kept those interactors that were present in LLPS databases because they have been published to undergo LLPS in vitro or in vivo [27–30]. We note that it is currently debated which proteins undergo LLPS in cells and how to experimentally confirm this. We focused here on candidate proteins included in databases that reflect the recent literature, with the intention to consider scenarios discussed in the community. We have also developed an interactive tool to consider alternative sets of candidate scaffolds (Additional files 2–6). Next, we used published quantitative mass spectrometry data [31] to obtain the copy numbers of the respective proteins in mESC lines. To estimate the fraction of each candidate protein in the subnuclear organelle, we retrieved the volumes of the organelle and the nucleus, and then estimated the enrichment of the respective protein in the organelle based on published microscopy images that visualize it by immunostaining or by fusion to a fluorescent protein (Additional file 1: Tables S1–S3). Estimating enrichments from images seems compatible with the nano- to micromolar concentration range relevant for the proteins studied here (Additional file 1: Fig. S2), in which intensities are proportional to concentrations (for more details, see “Methods” section). We note that both fixation and permeabilization prior to immunostaining and expression of fusion proteins may change the observed enrichment, introducing some uncertainty at this step. We next determined the RNA content of the organelle based on the known net amount [32, 33] and spatial distribution [34, 35] of nuclear RNA, and we also estimated its nucleosome content based on the total number of nucleosomes and their spatial distribution. The entire workflow, which is further detailed in the “Methods” section, yields a molecular census of an organelle of interest, serving as a basis to estimate average intermolecular distances.

### The molecular composition of prominent subnuclear organelles correlates with their activity

**Fig. 2:**
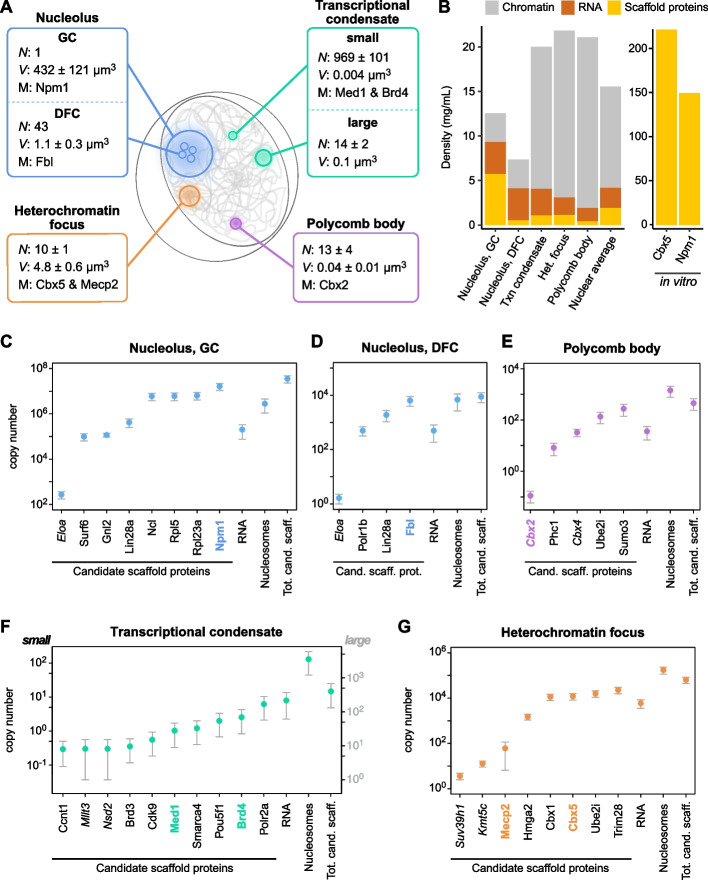
Molecular census of prominent membraneless organelles in the cell nucleus. **A** Overview of the subnuclear organelles studied here, including their volumes, numbers and marker proteins. **B** Mass densities of different classes of macromolecules, namely candidate scaffold proteins, RNAs, and chromatin (nucleosomes and linker DNA), in subnuclear organelles (left) and reconstituted condensates (right). Note that proteins that are not considered as candidate scaffolds are not included, so that the values do not add up to the total mass fraction of the organelles. **C**–**G** Estimated copy numbers of molecules in subnuclear organelles. The left and right *y*-axis for transcriptional condensates indicates copy numbers in small and large transcriptional condensates, respectively

We studied four membraneless organelles in the cell nucleus that can be observed in living and fixed cells and that have been suggested to form via phase separation (Fig. 2A, Additional file 1: Table S1): (i) Nucleoli, the sites of ribosome biogenesis that contain ribosomal DNA, transcribed by RNA Polymerase I, along with hundreds of different protein species [36]. They are partitioned into multiple subdomains [36–38], including the dense fibrillar component (DFC) and the granular component (GC), which are marked by fibrillarin (Fbl) and nucleophosmin (Npm1), respectively [39, 40]. (ii) Transcriptional condensates at active genes transcribed by RNA Polymerase II, which contain coactivators and components of the transcription machinery [41–43], including the Mediator complex with its subunit Med1, as well as Bromodomain-containing protein 4 (Brd4) [43]. Transcriptional condensates in mouse ESCs have been divided into two subpopulations with different sizes [42]. (iii) Heterochromatin foci containing silenced pericentric repeats marked with DNA methylation and di/trimethylation at lysine 9 of histone H3 (H3K9me2/3) [44, 45], which is recognized by heterochromatin proteins including Mecp2 and Heterochromatin Protein 1 alpha (HP1α)/Chromobox 5 (Cbx5) [46, 47]. (iv) Polycomb bodies containing silent genes marked with monoubiquitylation at lysine 119 of histone H2A (H2AK119ub) and di/trimethylation at lysine 27 of histone H3 (H3K27me2/3), which are bound by Polycomb Group proteins [48, 49] including Chromobox 2 (Cbx2) [50, 51]. Polycomb bodies often localize at developmentally regulated genes and their protein composition can change among cell types, suggesting that their properties may differ between mESCs and other cells.

To perform a molecular census of these organelles, we first determined the mass densities of candidate scaffold proteins, RNAs and nucleosomes (Fig. 2B). Nucleoli had the highest RNA content compared to the other organelles, while heterochromatin foci and Polycomb bodies had the largest nucleosome content. For all organelles, the total mass density of candidate scaffold proteins, RNAs, and nucleosomes combined amounted to ~ 10–20 mg/mL. Considering that the total mass densities in subnuclear membraneless organelles of (differentiated) mouse cells lie between 45 and 100 mg/mL [52], these values suggest that the majority of the organelles studied here is filled with molecules that we did not classify as candidate scaffolds.

We next estimated the copy numbers of individual candidate scaffold proteins, RNA molecules, and nucleosomes (Fig. 2C–G, Additional file 1: Tables S2–S3). Each nucleolar DFC contains ~ 9000 candidate scaffold proteins, ~ 7000 nucleosomes, and ~ 500 RNA molecules. The larger nucleolar GC contains ~ 34 million candidate scaffold proteins, ~ 2.7 million nucleosomes, and ~ 200,000 RNA molecules. Thus, the GC contains an excess of candidate scaffold proteins over nucleosomes and RNA molecules, which might seem surprising given that nucleoli are highly enriched in RNA [34]. However, cells contain globally more proteins than RNAs [53], only a relatively small fraction of RNAs localize to the nucleus [33], and nucleoli contain a plethora of proteins in addition to the ribosomal proteins whose number equals roughly that of ribosomal RNAs [53]. Our estimates for small transcriptional condensates suggest that they contain only a few molecules, i.e., ~ 6 RNA Polymerase II molecules, ~ 3 Brd4 molecules, and ~ 1 Med1 molecule each, along with ~ 8 RNA molecules and ~ 130 nucleosomes (Fig. 2F, left axis). These numbers suggest that small transcriptional condensates may correspond to individual active genes with their enhancers, which are bound by few transcription factors and a Mediator complex. Large transcriptional condensates contain hundreds of candidate scaffold proteins and RNA molecules as well as thousands of nucleosomes (Fig. 2F, right axis), which translates into tens of active genes with their (super)enhancers. While the prototypic small and large transcriptional condensates considered here represent prominent populations found in the cell, there might also be transcriptional condensates with intermediate sizes and copy numbers.

The two transcriptionally silent organelles contain an excess of nucleosomes (Fig. 2E and G, Additional file 1: Table S2). Each heterochromatin focus contains ~ 180,000 nucleosomes or ~ 33 Mb of DNA, corresponding to ~ 330 Mb of DNA in all the 10 heterochromatin foci combined. This compares to the expected ~ 230 Mb of DNA in heterochromatin foci of diploid cells, considering a haploid genome size of 2.5 Gb [54], a major satellite content of ~ 3% in the genome [55], and a major satellite content of ~ 66% in heterochromatin foci [56]. The most abundant candidate scaffold protein in heterochromatin is Trim28, with a copy number of ~ 23,000 per focus. The HP1 paralogs Cbx1 and Cbx5 are less abundant, each with a copy number of ~ 12,000 per focus. Accordingly, less than 10% of the nucleosomes are bound by a given HP1 paralog. The copy numbers of Cbx1/Cbx5 translate into concentrations of ~ 4 μM (Additional file 1: Table S3), which is similar to the values experimentally obtained in mouse fibroblasts [47]. Other prominent heterochromatin proteins like Mecp2 [57], Kmt5c [58], and Suv39h1 [59] are much less abundant (Fig. 2G,Additional file 1:Table S3). For Polycomb bodies, we estimated a chromatin content of ~ 1400 nucleosomes or ~ 260 kb of DNA (Additional file 1: Table S2), with the PcG proteins Cbx2 and Cbx4 being very lowly abundant (Fig. 2E, Additional file 1: Table S3). Both types of transcriptionally silent organelles contain relatively low RNA levels, with concentrations of approximately 0.2–0.3 μM.

### Homotypic protein self-interactions are unlikely to drive PSCP/LLPS in subnuclear organelles

**Fig. 3:**
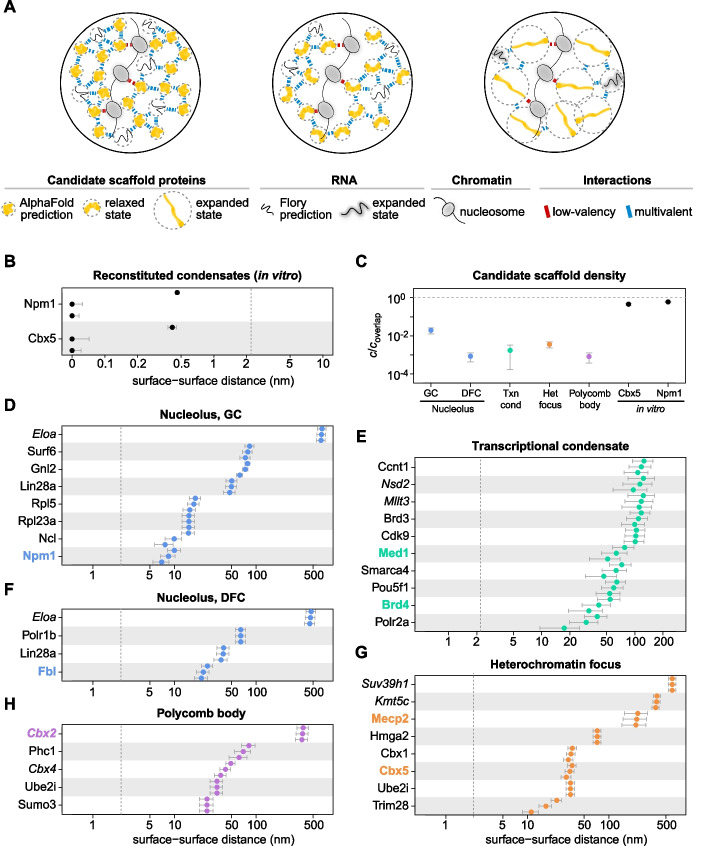
Distances between proteins of the same type are incompatible with phase separation driven by homotypic self-interactions. **A** Scenarios used to determine intermolecular distances. We considered predicted (left), relaxed (center) and expanded (right) conformations. **B** Distances between surfaces of neighboring proteins in condensates that were reconstituted with purified proteins in vitro. Distances for predicted (top), relaxed (center), and expanded (bottom) conformations are shown. The dashed line indicates the estimated maximum Debye length in cells. **C** Density of candidate scaffold proteins in subnuclear organelles and reconstituted condensates in vitro. Values are indicated with respect to the concentration at which proteins overlap if they adopt their predicted conformation. **D**–**H** Distances between the surfaces of neighboring proteins of the same type in subnuclear organelles, considering predicted (top), relaxed (center) and expanded (bottom) conformations. The dashed lines indicate the estimated maximum Debye length in cells

We next estimated intermolecular distances in each subnuclear organelle. We converted copy numbers into concentrations (Additional file 1: Table S3) and into average distances among the surfaces of neighboring molecules (see “Methods” section). To do so, we assumed that each molecule can explore a spherical volume whose radius corresponds to the molecule's radius of gyration, and we assumed a random spatial distribution of molecules within the organelle (Fig. 3A). Pronounced spatial inhomogeneities in the organelle could therefore change our estimates. Radii of gyration for proteins were obtained from AlphaFold [60], a resource for predicted protein structures. To account for the flexibility of disordered regions and their propensity to adopt extended conformations [61], whose sizes might be underestimated by AlphaFold, we also considered two larger sizes (Additional file 1: Table S4). We refer to them as “relaxed” and “expanded” conformations (see “Methods” section). Sizes of nucleosomes and RNAs were obtained from the crystal structure [62] and a published scaling law [63], respectively.

We first determined intermolecular distances for proteins whose concentration in the dense phase has been experimentally measured in vitro (Fig. 3B). We calculated three intermolecular distances for each protein, using the three molecular sizes (predicted, relaxed, expanded) introduced above. For both proteins, the resulting distances between the surfaces of neighboring molecules were below the Debye length, which is ~ 0.8 nm in a solution of physiological ionic strength [8]. This confirms that molecules in these condensates are close enough to each other to interact via multivalent homotypic self-interactions. Consistently, the concentration in these condensates is close to the so-called overlap concentration (Fig. 3C), which is an alternative measure of the density of a macromolecular solution (see “Methods” section). Next, we determined the distances between surfaces of candidate scaffold molecules in cellular organelles (Fig. 3D–H). We calculated distances for each candidate scaffold protein species on its own, to test the scenario that the respective protein species undergoes one-component PSCP/LLPS via homotypic self-interactions. This is the scenario that has usually been considered when classifying proteins as LLPS scaffolds/drivers, and it has been tested in vitro for all the marker proteins above [40, 43, 46, 50, 51, 57]. Our analysis shows that all intermolecular distances for this scenario are well above the estimated Debye length, suggesting that none of the candidate scaffold proteins can establish multivalent homotypic self-interactions in its organelle because neighboring molecules of the same type are too far apart from each other. Due to these large distances, PSCP/LLPS based on homotypic self-interactions is unlikely to play a major role in stabilizing the respective subnuclear organelles.

### RNA may enable multicomponent PSCP/LLPS in transcriptionally active organelles

**Fig. 4:**
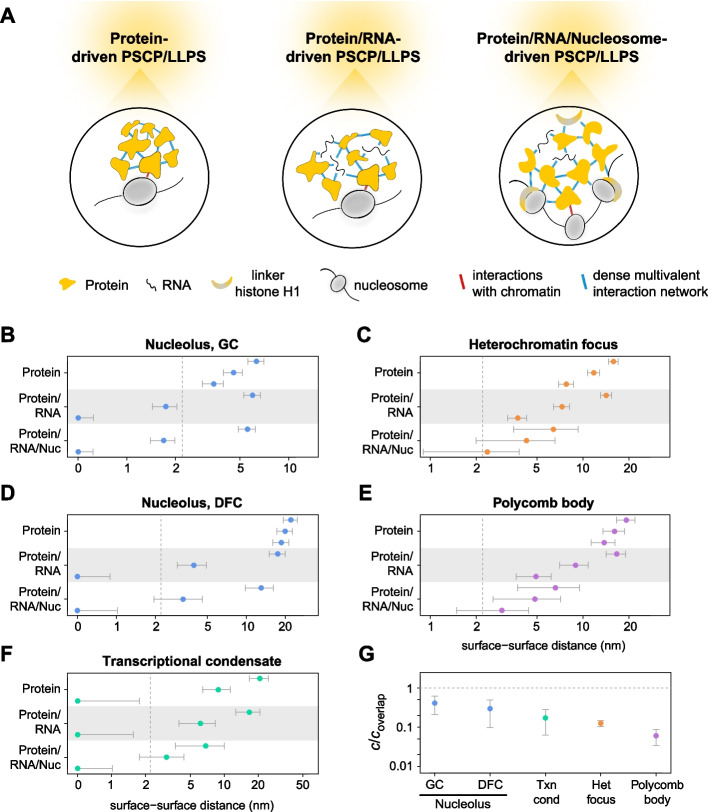
Distances between candidate scaffolds of all species are compatible with multicomponent phase separation in RNA-rich transcriptionally active organelles. **A** Scenarios considered for multicomponent condensates. Either all candidate scaffold proteins (left), all candidate scaffold proteins and RNAs (center) or all candidate scaffold proteins, all RNAs and all nucleosomes (right) establish multivalent interactions with each other. **B**–**F** Distances between neighboring molecules of the indicated types in subnuclear organelles. The dashed lines indicate the estimated maximum Debye length. Distances for predicted (top), relaxed (center), and expanded (bottom) conformations are shown. Intermolecular distances in nucleoli and transcriptional condensates are below the estimated Debye length if all candidate scaffold proteins and RNAs participate in the multivalent interaction network and if relaxed or expanded conformations are considered. **G** Density of candidate scaffold proteins, RNAs, and chromatin in subnuclear organelles. Values are indicated with respect to the concentration at which molecules overlap if they adopt their expanded conformation

We next considered the multicomponent scenario that assumes that different molecular species in the condensates establish multivalent interactions with each other, including homo- and heterotypic interactions. This scenario has become increasingly popular as it aligns better with the properties of membraneless organelles in the cell. However, for the organelles studied here, it has not been explicitly tested in experiments. It is generally not clear which scaffold proteins would co-condense into a common condensate instead of forming distinct condensates or multiphase condensates when being mixed together, and which impact the presence of client molecules would have. Another complication is our incomplete knowledge of scaffold proteins, as we might over- or underestimate their number when selecting them based on their propensity to undergo LLPS via homotypic interactions. In this context, a computational workflow can be useful to make predictions before engaging in cost- and labor-intensive experiments, which will be ultimately required to assess the impact of the biochemical and biophysical properties of the respective molecules on condensate formation. With this motivation in mind, we studied the following multicomponent scenarios: (i) All candidate scaffold proteins considered above drive multicomponent PSCP/LLPS, (ii) all candidate scaffold proteins and RNAs drive multicomponent PSCP/LLPS, and (iii) all candidate scaffold proteins, RNAs, and nucleosomes drive multicomponent PSCP/LLPS (Fig. 4A). The latter implies the formation of molecular assemblies that are constitutively associated with chromatin, which may be understood as surface condensates [64]. These three scenarios we consider here are on the one hand optimistic, as they assume that all molecular species have compatible interaction surfaces and can form a joint percolating interaction network. They may on the other hand be pessimistic, as they only consider proteins in current LLPS databases that might be incomplete. To address the latter issue, we have also developed an interactive tool that can be used to consider different sets of candidate scaffold proteins (Additional files 2–6).

The intermolecular distances obtained for the three above-mentioned scenarios are shown in Fig. 4B–F. Intermolecular distances in nucleoli, heterochromatin foci and Polycomb bodies are beyond the estimated Debye length if RNAs and nucleosomes do not participate in driving PSCP/LLPS. Intermolecular distances in transcriptional condensates are also beyond the estimated Debye length if folded and relaxed conformations are considered, but become smaller than the estimated Debye length if expanded conformations are used. In the multicomponent scenario for transcriptional condensates, we considered the entire Mediator complex and the entire RNA Polymerase II elongation complex as scaffolds, assuming that most Med1 and RNA Polymerase II molecules are present in these complexes, which contain several disordered protein stretches and exhibit multiple interaction surfaces [65, 66]. For the scenario of expanded conformations, we also increased the sizes of these complexes. It is unclear how appropriate this assumption is, as the structure of both complexes has to our knowledge not been studied within condensates. Furthermore, we did not consider any of the most prominent RNA-binding proteins in the calculation because none of them was identified as candidate scaffold by our workflow. However, nascent transcripts produced by RNA Polymerase II associate with multiple RNA-binding proteins, and many of them are disordered and can undergo PSCP/LLPS [67]. It seems therefore likely that transcriptional condensates contain enough scaffolds that are close enough to each other to interact, especially if RNA-bound proteins are included. If RNA molecules themselves are also considered as scaffolds, intermolecular distances in nucleoli become smaller than the estimated Debye length, while intermolecular distances in heterochromatin foci and Polycomb bodies remain larger than the estimated Debye length. Consistent with these results, the total scaffold concentrations are lower in heterochromatin foci and Polycomb bodies than in nucleoli and transcriptional condensates (Fig. 4G). If nucleosomes are considered as additional scaffolds, intermolecular distances in heterochromatin foci and Polycomb bodies remain still above the estimated maximum Debye length but get close to it if expanded conformations are considered.

Taken together, when considering the candidate scaffold proteins in current LLPS databases, our results suggest that transcriptionally active organelles are compatible with multicomponent PSCP/LLPS if RNA acts as a scaffold and if scaffolds adopt relaxed (nucleolar GC) or extended (nucleolar DFC and transcriptional condensates) conformations. The model that RNA is required for a sufficiently dense interaction network fits to the observations that nucleoli and small transcriptional condensates are sensitive to the inhibition of RNA polymerase I and II, respectively [42, 68]. Transcriptionally inactive organelles exhibit larger intermolecular distances and are better compatible with ICBS than with PSCP/LLPS, although a multicomponent surface condensation scenario cannot be completely ruled out, which would require proteins and RNAs to adopt expanded conformations and nucleosomes to participate in the multivalent interaction network. Notably, we did not consider the polymer–polymer phase separation scenario in the present study, which might also play a role in the biogenesis of the above-mentioned organelles.

### A scoring system elucidates the demixing mechanism of subnuclear organelles

**Fig. 5:**
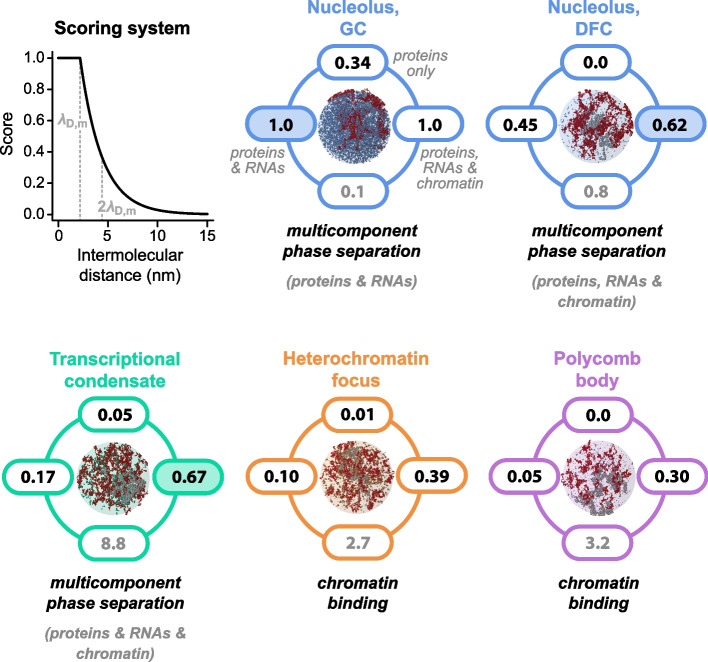
Cells likely use different demixing mechanisms to assemble their subnuclear organelles. We used a scoring system (top left) to translate intermolecular distances into predictions of the most likely demixing mechanism in the form of scores (0: unlikely; 1: likely). λ_D,m_ denotes the estimated maximum Debye length of 2.2 nm. The molecular composition and the most likely demixing mechanism is depicted for the different membraneless organelles studied here. For each of them, virtual microscopy images, scores (black numbers) and nucleosome-to-protein ratios (gray numbers) are shown. In virtual microscopy images, nucleosomes are shown in gray, RNAs in dark red, and candidate scaffold proteins in other colors (molecules are drawn to scale). Depicted volumes correspond to one pixel of a virtual confocal microscopy image

We developed a scoring system to visualize the predicted demixing mechanism (Fig. 5, top left). For each organelle, it scores intermolecular distances (black numbers) considering only proteins (top), proteins and RNAs (left), and proteins, RNAs, and nucleosomes (right). High scores reflect small intermolecular distances and a high likelihood for multivalent interactions among the respective classes of molecules to drive PSCP/LLPS-type phase separation. Low scores have the opposite meaning, and three low scores indicate that organelles are most likely formed by an alternative mechanism, such as ICBS. Nucleoli and transcriptional condensates feature high scores if proteins, RNAs, and potentially nucleosomes are included (Fig. 5), indicating that both organelles are in line with multicomponent PSCP/LLPS. Heterochromatin foci and Polycomb bodies exhibit lower scores, indicating that these organelles are formed by ICBS rather than PSCP/LLPS driven by the scaffold proteins considered here. Ratios between candidate scaffold proteins and nucleosomes are also indicated for each organelle (gray numbers, bottom), showing that heterochromatin foci and Polycomb bodies exhibit an excess of nucleosomes that can serve as binding sites in an ICBS scenario. We would like to note that both PSCP/LLPS and ICBS may go along with a phase transition at the level of the nucleosome chain, potentially giving rise to polymer–polymer phase separation [11, 15, 16], which is a possibility we did not address here.

Based on our molecular census, we generated virtual microscopy images of the individual organelles to illustrate the stoichiometries and sizes of their constituents (Fig. 5). Each image shows a spherical volume element with a radius of 250 nm, roughly corresponding to the observation volume of a confocal microscope. Candidate scaffold proteins in transcriptionally silent organelles (orange/purple) are far apart from each other and do not form any visible connected network alone or with RNA (dark red) and nucleosomes (gray). In transcriptionally active organelles, a percolating network spanning the organelles can be discerned, which is a hallmark of PSCP/LLPS.

## Discussion

Here, we present a molecular census of subnuclear membraneless organelles that is based on the host of public datasets available for mouse embryonic stem cells. We use it to estimate the numbers and intermolecular distances of molecules in prominent membraneless organelles, making it possible to test the compatibility of different demixing mechanisms with simple laws from molecular biophysics. Our workflow is open-source and can be adapted to study any cellular organelle in any cell type for which the required datasets are available.

We found that none of the candidate scaffold proteins in the organelles studied here fulfills the conditions to undergo phase separation via homotypic self-interactions, which is the scenario that has commonly been considered. Intermolecular distances are well-above the estimated Debye length in cells, so that the members of a single protein species appear to be unable to form the dense multivalent interaction network that drives PSCP/LLPS. This conclusion is in line with a recent study that has questioned the predictive value of homotypic interactions for subcellular localization [69]. In stark contrast, our estimates for reconstituted condensates in the test tube yield intermolecular distances below the Debye length, validating our approach and highlighting the differences between membraneless organelles in cells and reconstituted condensates assembled in vitro.

When considering the scenario that multiple components come together to form condensates via heterotypic multivalent interactions, we found that the molecular composition of transcriptionally active organelles is in line with multicomponent phase separation, in which all candidate scaffold proteins, RNA molecules, and nucleosomes together are close enough to each other to form a dense multivalent interaction network, provided that scaffold proteins and RNAs adopt relaxed or expanded conformations [61]. Our analysis suggests that RNA molecules are a key ingredient of these condensates, which is in line with recent work [70]. The molecular composition of transcriptionally silent organelles, however, is less compatible with PSCP/LLPS-type phase separation, even if an unlimited ability of all components considered here to establish heterotypic multivalent interactions with one another is assumed. These organelles are well described by the ICBS model, in which chromatin-associated proteins act as clients that bind the chromatin scaffold without phase-separating. Conceptually, interactions between chromatin-associated proteins and the chromatin scaffold may alter the conformation of the latter and may potentially drive polymer–polymer phase separation, which is a scenario that we did not test in this work. Our conclusions about multicomponent phase separation are based on current LLPS databases. More work will be needed to establish criteria for the identification of scaffold proteins and to update and refine these databases. High-throughput experiments as well as artificial intelligence-based computational approaches may prove useful in this respect [71–73].

Our results, which integrate our current knowledge about nuclear condensates and their scaffolds, indicate that cells use different mechanisms to control membraneless organelles, favoring PSCP/LLPS-type phase separation for active chromatin and ICBS for silent chromatin. This view aligns well with several previous findings: PSCP/LLPS of synthetic scaffolds has been shown to occur preferentially in less compacted chromatin, i.e., outside of dense and silent heterochromatin structures [74]. Furthermore, transcriptionally active nucleoli show an interfacial barrier that is indicative of the dense interaction network underlying PSCP/LLPS, while transcriptionally silent mouse heterochromatin foci lack such a barrier [12, 75, 76]. A previous biochemical approach to purify *bona fide* phase-separated nuclear bodies has found that the latter are primarily associated with active chromatin regions, while they are depleted from silent repetitive heterochromatin [77]. Furthermore, RNAs have been found to play important roles in phase separation [70, 78, 79], e.g., by stabilizing and fluidizing condensates [80, 81]. The model that heterochromatin proteins undergo ICBS, i.e., that they bind largely independently to their genomic target sites, is in agreement with the finding that depletion of one of these proteins does not affect the localization of the others and does not perturb heterochromatin integrity, which has for example been shown for HP1α/Cbx5 and Mecp2 [82], two prototypic markers of heterochromatin foci. In contrast, depletion of Npm1 or rRNA (via inhibition of RNA Polymerase I) disrupts the structural integrity of nucleoli [68, 83], which is in agreement with our predictions and the notion that the nucleolus is a multicomponent condensate with mutual interdependence among its components.

## Conclusions

We developed a by-the-numbers approach to phase separation to complement bottom-up simulation schemes as well as experimental assays studying phase separation either with recombinant proteins in the test tube or with fluorescently labeled proteins in living cells. It can naturally evolve to take into account data that will be generated in the future, e.g., updated selections of scaffold proteins or novel information about their conformations in condensates. Based on our current knowledge about the selected organelles in mouse embryonic stem cells, a popular model system for mammalian biology, we propose that these cells contain different classes of membraneless organelles that are formed by different biophysical mechanisms. This may allow cells to create separated microenvironments for dedicated cellular activities where needed, e.g., for the production and processing of ribosomal RNAs and for the repair of damaged DNA, while using the default nucleoplasmic environment as a reaction medium for other activities. More broadly, our work highlights the links between different demixing mechanisms and the internal nanoscale features of the resulting membraneless organelles. We hope that our work will stimulate experiments to assess features of subnuclear organelles that are currently only partially characterized but prove crucial to understand demixing, including the exact Debye length in cells as well as the spatial distributions, conformational ensembles, and co-condensation behavior of scaffold molecules. We also hope that our work will inspire models that assess the interplay between different demixing mechanisms that likely occur with overlapping sets of proteins in the same cell nucleus side-by-side.

## Methods

### Identification of candidate scaffold proteins

To identify candidate scaffold proteins in a membraneless organelle of interest, we first selected a prominent marker protein that is present in the organelle. Subsequently, we identified interactors of the marker protein using the STRING database [26]. The default parameters for the query function of the R package STRINGdb were used [26]. Among the resulting proteins, we retained those that are annotated in at least one of the databases of LLPS scaffold/driver proteins considered here [27, 28, 30]. We also conducted a literature search to retain proteins that have recently been shown to phase-separate in vitro and have not yet been entered in the above-mentioned databases. Furthermore, we added proteins that have been reported to be present in an organelle of interest and have been shown to potentially undergo LLPS, but that were not picked up as interactors in STRINGdb. We removed proteins that are known to function only in special cases (e.g., mitosis, hormone stimulation). The resulting candidate scaffold proteins along with their abundance are listed in Additional file 1: Table S3.

### Total protein copy numbers per cell

Mass spectrometry data from mESCs were taken from [31]. The iBAQ values reported in two different replicates were averaged and converted into numbers of molecules per cell according to the following strategy:

First, the copy numbers of CTCF and Rad21, which have been experimentally measured in mESCs using a combination of “in-gel” fluorescence, fluorescence correlation spectroscopy (FCS)-calibrated imaging, and flow cytometry, were retrieved [84]. Furthermore, the copy numbers of PRC1 proteins Ring1b and Pcgf6 as well as Rybp measured by fluorescence microscopy and “in-gel” fluorescence were retrieved [85]. Second, the theoretical number of histone H4 molecules was calculated according to

1 $${N}_{\text{H4,tot}}=4\cdot \frac{2.5\cdot {10}^{9}\, \text{bp}}{186\, \text{bp}}\approx 54\cdot {10}^{6}$$

This expression is based on a haploid genome size of 2.5 Gb [54], a nucleosome repeat length of 186 bp [86], a ploidy of 2n (diploid), and the presence of two H4 molecules per nucleosome.

Using the copy numbers for CTCF, Rad21, Ring1b, Pcgf6, Rybp, and H4, a linear regression was used to establish a relationship between both quantities according to:

2 $${N}_{i,\text{tot}}=a\cdot {\text{iBAQ}}_{i}$$

Using the resulting scaling parameter *a*, the iBAQ value of each protein of interest was converted into the copy number per cell. Proteins that were not detected in the mass spectrometry experiment were assigned a copy number of 407, which corresponds to the detection limit estimated from the proteome-wide copy number distribution.

As shown in Additional file 1: Fig. S3, the copy numbers of the candidate scaffold proteins of interest determined here are in good agreement with those reported in an independent study [87].

### Protein copy numbers and concentrations in organelles

To determine copy numbers in organelles (*N*_org_) based on the total copy numbers per cell (*N*_tot_), the cellular protein pool was decomposed into two pools that correspond to proteins inside and outside of organelles:

3 $${N}_{\text{tot}}={N}_{\text{in}}+{N}_{\text{out}}={{{n}_{\text{org}}V_{\text{org}}}c_{\text{org}}}+{{V}_{\text{np}}c_{\text{np}}}={{{n}_{\text{org}}V_{\text{org}}}c_{\text{org}}}+{\left({{V}_{\text{nuc}}-{n}_{\text{org}}V_{\text{org}}}\right)c_{\text{np}}}$$

Here, *V*_org_, *V*_np_, and *V*_nuc_ are the volumes of one of the organelles of interest, the nucleoplasm and the nucleus, respectively; ${c}_{\text{org}}={N}_{\text{org}}/{V}_{\text{org}}$ and ${c}_{\text{np}}={N}_{\text{np}}/{V}_{\text{np}}$ are the average concentrations in the organelle and the nucleoplasm, respectively; and *n*_org_ is the number of organelles per nucleus. Note that all organelles considered here are located in the nucleus. The expressions can easily be adapted for cytoplasmic organelles. If proteins that do not exclusively localize to the nucleus are considered, the cytoplasmic pool has to be taken into account, too.

Having determined *N*_tot_ from mass spectrometry experiments as described above, and having determined the volume of the nucleus and the organelle as well as the number of organelles based on the literature (Additional file 1: Table S1), the only unknown variables in Eq. 3 are the average concentrations ${c}_{\text{org}}$ and ${c}_{\text{np}}$. Their ratio can be obtained from microscopy images in which the protein of interest is labeled, unless the dye/fluorophore concentration is too high so that there is no linear relationship between concentration and fluorescence intensity (due to quenching and absorbance effects). For concentrations up to tens of micromolar, a linear relationship has been found for various dyes as well as for GFP [76, 88–91], while a non-linear regime is reached for higher concentrations [76, 92]. The concentrations of the proteins considered here are generally small enough to fall into the linear regime (Additional file 1: Fig. S2), in particular if not every protein is labeled.

To quantify intensity ratios, organelles and nucleoplasm are segmented, and the average intensities *I*_org_ and* I*_np_ are quantified. Note that this workflow does not make any assumptions about the distribution of the protein of interest within the organelle or within the nucleoplasm. The average concentrations are only defined for mathematical convenience, and the image analysis workflow is essentially used to determine the net intensity inside and outside of the organelles, which scales with the respective total numbers of molecules divided per volume. Accordingly, we introduce the enrichment $\alpha$, which gives the ratio between both quantities:

4 $$\alpha =\frac{{I}_{\text{org}}-{I}_{\text{BG}}}{{I}_{\text{np}}-{I}_{\text{BG}}}=\frac{{c}_{\text{org}}}{{c}_{\text{np}}}$$

Here, ${I}_{\text{BG}}$ corresponds to the background intensity of the images that we subtracted. Combining this expression with the one above and solving for ${c}_{\text{org}}$ yields

5 $${c}_{\text{org}}=\frac{{\alpha N}_{\text{tot}}}{{\left(\alpha -1\right){ n}_{\text{org}}V_{\text{org}}}+{V}_{\text{nuc}}}$$

Finally, the copy number in one organelle of interest is obtained via

6 $${N}_{\text{org}}={c}_{\text{org}}{V}_{\text{org}}=\frac{{\alpha N}_{\text{tot}}{V}_{\text{org}}}{{\left(\alpha -1\right) {n}_{\text{org}}V_{\text{org}}}+{V}_{\text{nuc}}}$$

By determining all parameters on the right-hand site of the last two expressions, the copy numbers and concentrations in the organelle are obtained.

For a few proteins, we did not find published microscopy images in mouse ESCs. For simplicity, we assumed for most of them that they exclusively localize to the organelle of interest ($\alpha =\infty$). This affected mostly lowly abundant proteins (Additional file 1: Table S3, proteins with $\alpha =\infty$) and is therefore not critical for our conclusions.

In the case of proteins for which we found published microscopy images, we assumed that the bright regions in the image correspond to the organelle of interest. This is typically backed up by the literature about the respective proteins. For some cases, for which we found images in which both the protein of interest and a marker for the respective organelle was labeled, we segmented the organelle based on the marker and measured the intensity of the protein of interest within the organelle based on this segmentation. As heterochromatin foci and nucleoli are visible as regions with high and low signal in DAPI stains, respectively, such stains were used as markers where applicable.

### RNA copy numbers and concentrations in organelles

The RNA content of organelles was determined with a similar strategy as the protein content (described above). First, the mass of RNA in the nucleus was determined based on the total mass of cellular RNA (*M*_tot_ = 20 pg) and the fraction of nuclear RNA versus total RNA (*f*_nuc_ = 15%), according to published data [32, 33]. The mass of nuclear RNA was then converted into the number of ribonucleotides in the nucleus:

7 $${N}_{\text{rn},\text{tot}}=\frac{{M}_{\text{tot}}{ f_{\text{nuc}}}}{MW}$$

Here, *MW* is the molecular weight of a ribonucleotide (340 g/mol for a ribonucleotide monophosphate).

Next, images showing the spatial distribution of RNAs [34, 35] were used to calculate the RNA enrichment in the organelle of interest. To this end, heterochromatin foci and nucleoli were segmented based on their high and low signal in DAPI stains [34, 35], respectively, and the integrated RNA signal was quantified for each of them. The RNA signal in the nuclear space that surrounds heterochromatin foci and nucleoli, which contains transcriptional condensates and Polycomb bodies, appeared quite homogenous, which is why we used an RNA enrichment of unity for these organelles. Accordingly, the nuclear RNA pool can be decomposed according to:

8 $${N}_{\text{rn},\text{tot}}={N}_{\text{rn},\text{np}}+\sum_{i}{N}_{\text{rn},\text{org},i}={c}_{\text{rn},\text{np}}\left({V}_{\text{np}}+\sum_{i}{{\alpha }_{\text{org},i} V_{\text{org},i}}\right)$$

Here, ${N}_{\text{rn},\text{np}}$ and ${c}_{\text{rn},\text{np}}$ are the number and concentration of ribonucleotides in the nucleoplasm, respectively, ${V}_{\text{np}}$ is the volume of the nucleoplasm, and ${N}_{\text{rn},\text{org},i}$, ${V}_{\text{org},i}$, and ${\alpha }_{\text{org},i}$ are the number of ribonucleotides, the volume, and the RNA enrichment in the *i*th organelle, respectively.

Having determined the total number of ribonucleotides in the nucleus (${N}_{\text{rn},\text{tot}}$) as well as the enrichments ${\alpha }_{\text{org},i}$ and the volumes ${V}_{\text{org},i}$ and ${V}_{\text{np}}$ (see above and Additional file 1: Table S1), the concentration of ribonucleotides in the nucleoplasm is obtained according to:

9 $${c}_{\text{rn},\text{np}}=\frac{{N}_{\text{rn},\text{tot}}}{{V}_{\text{np}}+\sum_{i}{{\alpha }_{\text{org},i} V_{\text{org},i}}}$$

The number of ribonucleotides in the organelles is then obtained via:

10 $${N}_{\text{rn},\text{org},i}={c}_{\text{rn},\text{org},i} {V}_{\text{org},i}={{c}_{\text{rn},\text{np}} \alpha _{\text{org},i}} {V}_{\text{org},i}=\frac{{{\alpha }_{\text{org},i} V_{\text{org},i}}{ N}_{\text{rn},\text{tot}}}{{V}_{\text{np}}+\sum_{k}{{\alpha }_{\text{org},k} V_{\text{org},k}}}$$

Here, ${c}_{\text{rn},\text{org},i}$ is the concentration of ribonucleotides in the *i*th organelle. To convert the number of ribonucleotides into the number of RNA molecules, the former is divided by the respective RNA length, which corresponds to ~ 2790 nt for mRNA or ~ 14,000 nt for (precursor) rRNA [54, 93]. We used the latter value for nucleoli and the former value for the other organelles.

### Nucleosome numbers and concentrations in organelles

The number of nucleosomes in each organelle was obtained similar to the number of scaffold proteins described above, using the total number of histone H4 molecules in the cell (see above), the fact that each nucleosome contains two H4 molecules [62], and the spatial distribution of chromatin in the cell as observed in DAPI stains [94]. The value for heterochromatin foci, which contain AT-rich satellite sequences that are preferentially stained by DAPI, was divided by 1.5 to account for the preference of DAPI to bind these sequences. We have previously determined this preference by comparing the DAPI signal to that of RFP-tagged histones in the same cells [47].

### Sizes of proteins, RNAs, and nucleosomes

To determine the size of candidate scaffold proteins, we obtained their predicted 3D structure from AlphaFold [60] and calculated their radius of gyration using the R package Bio3d [95]. The radii of gyration were multiplied by a factor of 2 to obtain diameters ${d}_{\text{protein},\text{AlphaFold}}$ (Additional file 1: Table S4). We consider proteins to explore distances corresponding to their radius of gyration around their center of mass to establish multivalent interactions with neighbors.

For the more extended conformations that proteins might adopt within biomolecular condensates, we partitioned each protein into ordered and disordered domains based on a secondary structure prediction using “DSSP” in Bio3d [95, 96]. A window with a length of 5 amino acids was used to define the domains. The radii of gyration of the individual domains were then determined based on (i) the 3D structure from AlphaFold for the ordered domains, and (ii) the previously determined scaling law for intrinsically disordered proteins [97] for the disordered domains. Subsequently, two geometrical arrangements of the individual domains were considered: In the first one, which we refer to as “relaxed” conformation, the protein is represented as a random walk with step sizes corresponding to the sizes of the individual domains. The resulting diameter reads

11 $${d}_{\text{protein},\text{relaxed}}=2\sqrt{\sum_{i}{R}_{\text{g},\text{ordered},i}^{2}+\sum_{i}{R}_{\text{g},\text{disordered},i}^{2}}$$

If the resulting diameter was smaller than the value predicted by AlphaFold, we used the latter one for the relaxed scenario.

For the second arrangement, which we refer to as “expanded” conformation, we consider a conformation that lies between the relaxed conformation above and a conformation in which all domains are arranged along a straight line. The resulting diameter reads

12 $${d}_{\text{protein},\text{expanded}}=\frac{1}{2}\left({d}_{\text{protein},\text{straight}}+{d}_{\text{protein},\text{relaxed}}\right)=\left(\sum_{i}{R}_{\text{g},\text{ordered},i}+\sum_{i}{R}_{\text{g},\text{disordered},i}\right)+\sqrt{\sum_{i}{R}_{\text{g},\text{ordered},i}^{2}+\sum_{i}{R}_{\text{g},\text{disordered},i}^{2}}$$

For several proteins, the resulting diameter is larger than that of the fully denatured protein, which we calculated as a reference based on the respective scaling law [97]. In these cases, we used the diameter of the denatured protein for the expanded scenario.

The size of RNA molecules was estimated based on a previously published scaling law that describes the size and shape of published RNA structures [63]:

13 $${d}_{\text{RNA},\text{folded}}=2\cdot 0.55\cdot {N}^{0.33}$$

Here, *N* is the RNA length in nucleotides. We used a length of 14,000 nt for ribosomal RNA in nucleoli, and 2790 nt for the other organelles, which corresponds to the median mRNA length.

We also accounted for the possibility that RNA molecules can adopt a more expanded conformation, similarly to proteins. To this end, we described them as worm-like chains using the previously determined persistence length ${l}_{\text{p}}={0.15\cdot N}^{0.33}$ [63]. Accordingly, the size of expanded RNA molecules reads

14 $${d}_{\text{RNA},\text{expanded}}=2\sqrt{\frac{{l}_{\text{p}}\cdot {l}_{\text{c}}}{3}-{l}_{\text{p}}^{2}+2\frac{{l}_{\text{p}}^{3}}{{l}_{\text{c}}}(1-\frac{{l}_{\text{p}}}{{l}_{\text{c}}}(1-{e}^{-\frac{{l}_{\text{c}}}{{l}_{\text{p}}}}))}$$

Here, ${l}_{\text{c}}=0.696 \text{nm}\cdot N$ is the contour length of the RNA chain [98]. Furthermore, we considered an intermediate relaxed case

15 $${d}_{\text{RNA},\text{relaxed}}=\frac{1}{2}\left({d}_{\text{RNA},\text{folded}}+{d}_{\text{RNA},\text{expanded}}\right)$$

The volumes of proteins and RNAs were then obtained based on their diameters according to

16 $${V}_{\text{RNA}/\text{protein}}=\frac{4\pi }{3}{\left(\frac{{d}_{\text{RNA}/\text{protein}}}{2}\right)}^{3}$$

### Organelle density

The density of candidate scaffold molecules in an organelle of interest was obtained via

17 $$\frac{c}{{c}_{\text{overlap}}}=\frac{\sum_{i}{N}_{\text{org},i}{V}_{\text{scaffold},i}}{{V}_{\text{org}}}=\frac{{N}_{\text{tot}}}{{V}_{\text{org}}}\sum_{i}\frac{{N}_{\text{org},i}}{{N}_{\text{tot}}}{V}_{\text{RNA}/\text{protein},i}$$

Here, ${N}_{\text{org},i}$ and ${V}_{\text{RNA}/\text{protein},i}$ are the copy number and volume of the *i*th molecular species in the organelle, ${N}_{\text{tot}}$ is the total number of molecules in the organelle, and ${V}_{\text{org}}$ is the organelle volume. The combined overlap concentration for the system is ${c}_{\text{overlap}}={\left(\sum_{i}\frac{{N}_{\text{org},i}}{{N}_{\text{tot}}}{V}_{\text{RNA}/\text{protein},i}\right)}^{-1}$.

### Intermolecular distances

To determine intermolecular distances within organelles, we first calculated the volume that is available for each molecule. For organelles that contain a single molecular species, such as a droplet reconstituted with a single type of recombinant protein, this reads

18 $${V}_{1-\text{comp},\text{available}}=\frac{{V}_{\text{org}}}{{N}_{\text{org}}}=\frac{1}{{c}_{\text{org}}}$$

As introduced above, ${V}_{\text{org}}$, ${N}_{\text{org}}$, and ${c}_{\text{org}}$ are the volume of the organelle, the number of molecules in the organelle, and the concentration in the organelle, respectively. The latter is measured in number per volume, which corresponds to the molar concentration multiplied by the Avogadro constant. Concentrations within droplets have been measured for some in vitro systems as shown in Fig. 3B.

Based on the available volume and the assumption of a random distribution of molecules, the distance between the centers of neighboring proteins is obtained as follows [99, 100]:

19 $${\Delta }_{1-\text{comp},\text{center}}=2r+\frac{{e}^{8\phi } r}{3{\phi }^{1/3}}\Gamma \left(\frac{1}{3},8\phi \right)$$

Here, *r* is the radius of gyration of the molecule, $\phi =4 \pi {r}^{3}/{3 V}_{1-\text{comp},\text{available}}$ is its volume fraction, and $\Gamma$ is the incomplete Gamma function. We note that this expression is conceptually similar to others that have recently been used to estimate intermolecular distances in condensates and protein solutions based on the available volume [101, 102].

To obtain the distance between the surfaces of neighboring molecules, which is the distance over which intermolecular interactions act, we subtracted the diameter of the respective molecules:

20 $${\Delta }_{1-\text{comp},\text{surface}}={\Delta }_{1-\text{comp},\text{center}}-{d}_{\text{RNA}/\text{protein}}=\frac{{e}^{8\phi } r}{3{\phi }^{1/3}}\Gamma \left(\frac{1}{3},8\phi \right)$$

For multi-component organelles that contain more than one molecular species, we substituted the radius of gyration *r* in Eq. 20 by the weighted average

21 $${r}_{\text{avg}}=\sqrt[3]{\frac{\sum_{i}{N}_{i}{ \left(\frac{{d}_{\text{RNA}/\text{protein},i}}{2}\right)}^{3}}{\sum_{i}{N}_{i}}}$$

Here, ${N}_{i}$ and ${d}_{\text{RNA}/\text{protein},i}$ are the copy number and the diameter of the *i*th molecular species in the organelle, which are known from the analysis above. With this substitution, the distance ${\Delta }_{\text{multi}-\text{comp},\text{surface}}$ was obtained. For volume fractions $\phi \ge 0.65$, which is beyond the value for the random dense packing of rigid spheres [99], the intermolecular distance was set to zero.

### Scoring system

To summarize the properties of membraneless organelles in a compact way, we developed a scoring system. To represent intermolecular distances, we defined a score S_D_ according to

22 $$S_\text{D}(\Delta)=\left\{\begin{array}{rr}1,\vert&\Delta<\lambda_\text{D}\\e^{-(\Delta-\lambda_\text{D})/\lambda_\text{D}},\vert&\Delta\geq\lambda_\text{D}\end{array}\right.$$

Here, $\Delta$ is the intermolecular distance (considering either proteins alone, proteins and RNAs, or proteins, RNAs and nucleosomes) and ${\lambda }_{\text{D}}$ is the estimated maximum Debye length of 2.2 nm. To calculate $\Delta$, we used the relaxed protein conformations.

High S_D_ scores (${S}_{\text{D}}\to 1$) reflect scenarios in which neighboring molecules are close enough to each other to establish intermolecular interactions, which can drive PSCP/LLPS, while small S_D_ scores (${S}_{\text{D}}\to 0$) reflect scenarios in which neighboring molecules are too far apart from each other to interact. The cross-over occurs at the estimated maximum Debye length ${\lambda }_{\text{D}}$, which is the scale over which intermolecular interactions can be established, e.g., [8]. We used a cutoff of ${S}_{\text{D}}=0.5$ to reject or accept a scenario.

## Supplementary Information

Additional file 1: Supplementary Figures S1-S3 and Supplementary Tables S1-S4 [105–130].

Additional file 2. Web-based interactive molecular census for nucleolar GC.

Additional file 3. Web-based interactive molecular census for nucleolar DFC.

Additional file 4. Web-based interactive molecular census for transcriptional condensates.

Additional file 5. Web-based interactive molecular census for heterochromatin foci.

Additional file 6. Web-based interactive molecular census for Polycomb foci.

## Data Availability

Scripts can be accessed via GitHub [103] and Zenodo [104].
