# Supplementary material for: A molecular census to elucidate the demixing mechanism of membraneless organelles
Source: Genome Biol. 2025 Oct 9;26:347. doi: 10.1186/s13059-025-03806-0 (PMC12509355; doi:10.1186/s13059-025-03806-0)
Supplement: Supplementary file 1 — Additional file 1: Supplementary Figures S1-S3 and Supplementary Tables S1-S4 [105–130]. [file 13059_2025_3806_MOESM1_ESM.pdf]

Supplementary Figures

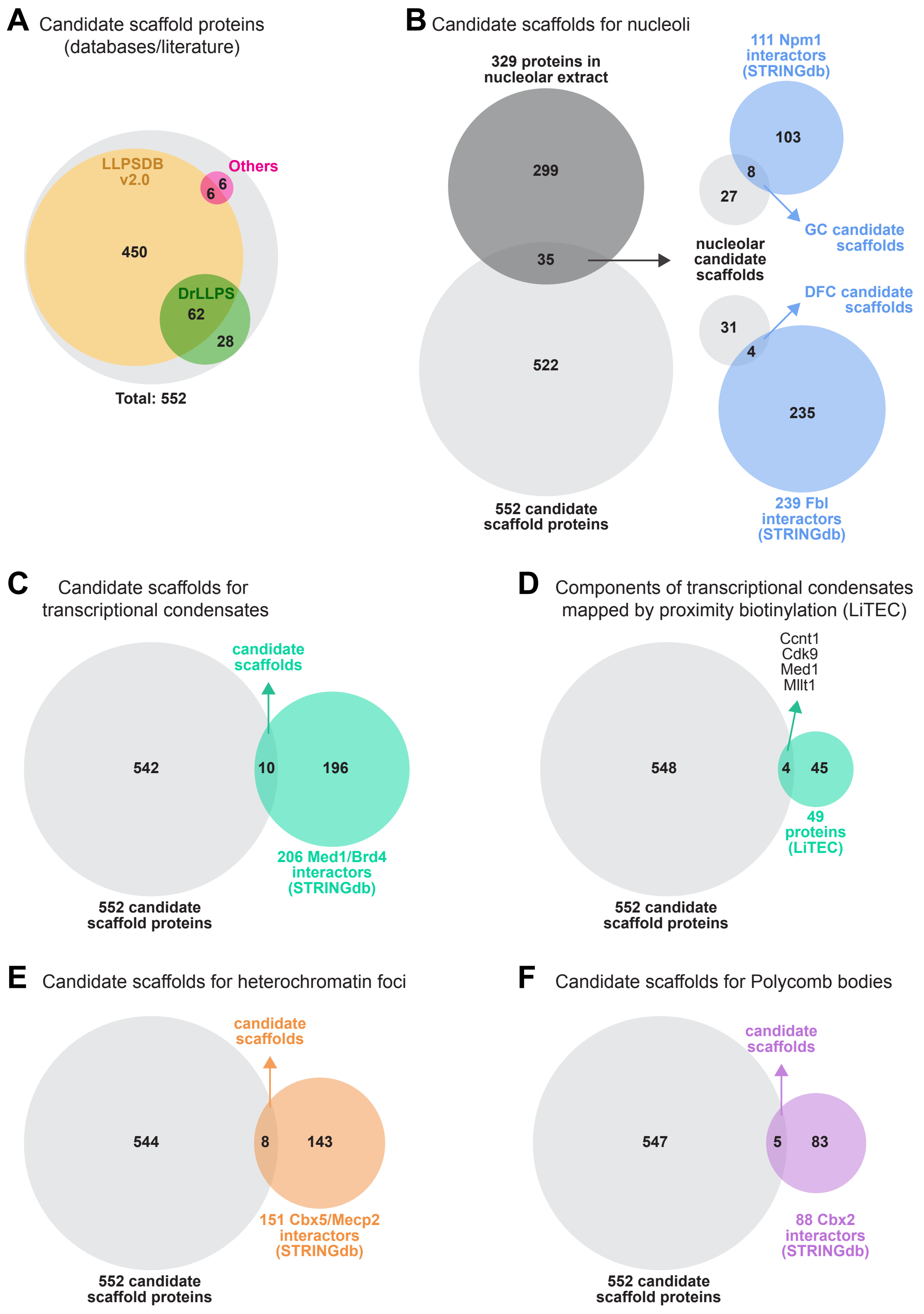

Figure S1. Workflow to identify candidate scaffold proteins. For comparison, the number of components of transcriptional condensates that were identified by light-induced proximity-based biotinylation [105] are shown in panel D.

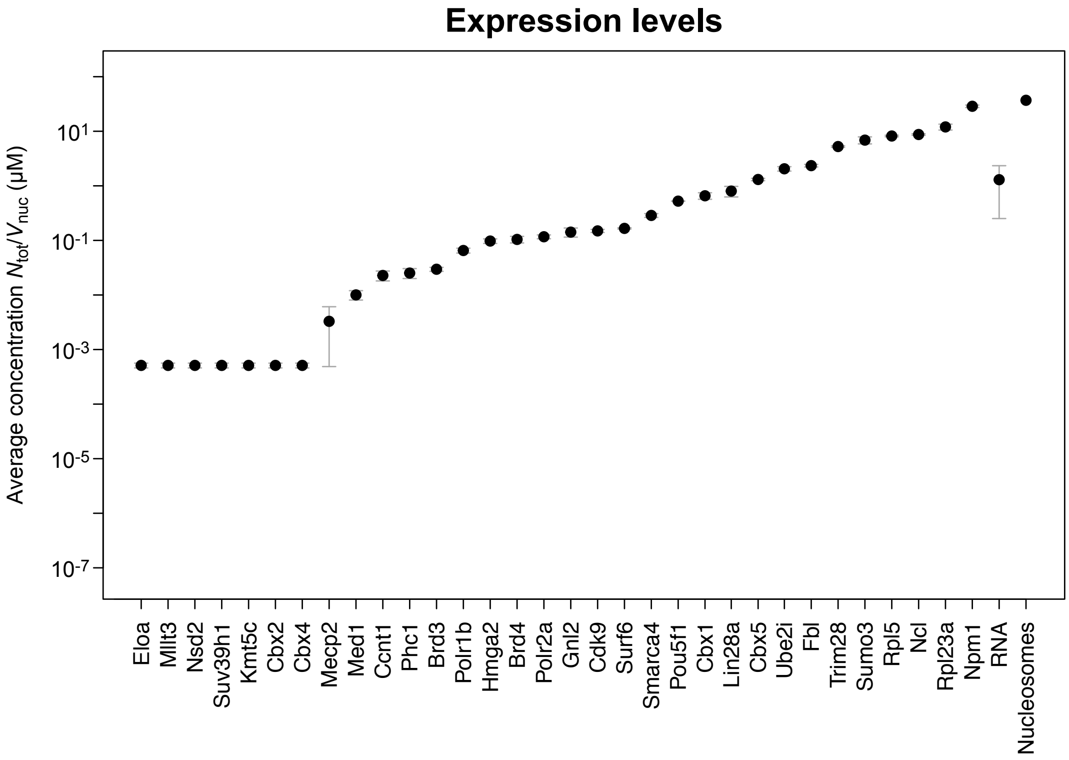

Figure S2. Expression levels of candidate scaffold proteins in the cell nucleus. Values are given as average concentrations that correspond to the copy number determined from mass spectrometry divided by the nuclear volume.

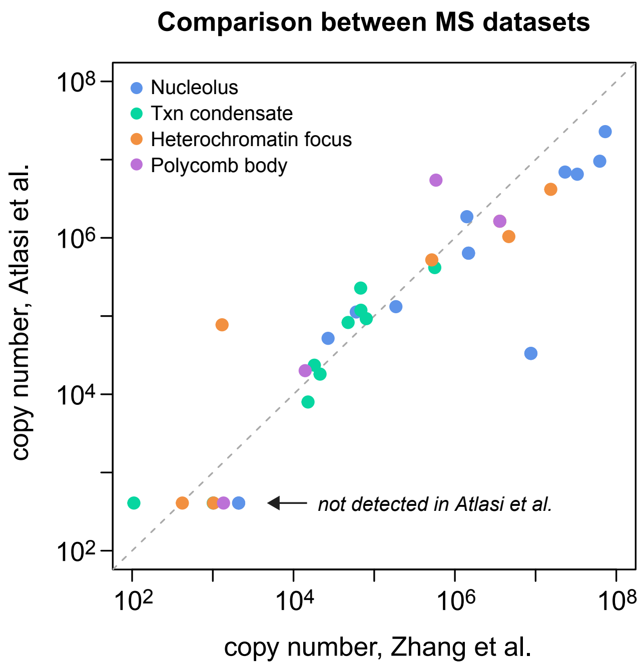

Figure S3. Copy numbers of candidate scaffold proteins in mouse embryonic stem cells based on two datasets from different studies [31, 87]. Copy numbers are color-coded according to the respective organelle as indicated.

Supplementary Tables

Organelle	Marker protein(s)	Volume (μm³)	Reference (volume)	Number per cell	Reference (number)
**Nucleus**	-	1320 ± 374	[106]	1	-
**Nucleolus, total**	-	480 ± 134	[106]	1	[107]
**Nucleolus, GC**	Npm1	432 ± 121	[106, 108]	1	[37]
**Nucleolus, DFC**	Fbl	1.1 ± 0.3		43	[37]
**Transcriptional condensate**	Med1, Brd4	0.004 (small)<br>0.1 (large)	[42]	983 ± 102 (total)	[43]
**Heterochromatin focus**	Cbx5, Mecp2	4.8 ± 0.6	[94]	10 ± 1	[94]
**Polycomb body**	Cbx2	0.04 ± 0.01	[51]	13 ± 4	[51]

Table S1. Cellular organelles studied in this work, along with their volume, number per mouse ES cell and the marker protein(s) that were used to identify them. GC, granular component; DFC, dense fibrillar component.

Organelle	Total number of scaffold proteins per organelle	Number of RNAs per organelle[a]	Number of nucleosomes per organelle	Reference (nucleo-somes)
**Nucleus**	62,496,930 ± 4,375,630	1,029,540 ± 829,688	29,348,434	-
**Nucleolus, GC**	34,181,482 ± 12,178,788	195,986 ± 121,723	2,711,831 ± 1,667,835	[109]
**Nucleolus, DFC**	8,926 ± 3,565	506 ± 317	7,007 ± 4,337	[109]
**Transcriptional condensate**	15 ± 10 (small)<br>400 ± 271 (large)	8 ± 6 (small)<br>216 ± 154 (large)	131 ± 86 (small)<br>3,535 ± 2,326 (large)	[43]
**Heterochromatin focus**	64,120 ± 19,979	6,028 ± 2,485	176,186 ± 59,805	[110]
**Polycomb body**	434 ± 207	35 ± 19	1,370 ± 629	[111]

[a] Note that RNA copy numbers in nucleoli refer to rRNA molecules, while RNA copy numbers in other organelles refer to shorter RNA molecules (median mRNA size). RNA copy numbers in the entire nucleus refer to a mixture of both types of RNA.

Table S2. Overview of the content of membraneless organelles studied in this work, according to the class of macromolecules. The total number of candidate scaffold proteins was determined based on quantitative mass spectrometry data for each candidate protein [31]. Note that the number indicated for the entire nucleus refers only to the candidates considered for the selected membraneless organelles studied here (not to all candidates for all membraneless organelles in the nucleus). The number of RNA molecules was determined based on the known amount of total RNA in the nucleus, the median length of different species of RNA molecules, and the spatial distribution of RNA molecules taken from stainings with an RNA-specific fluorescent probe (Extended Data Fig. 4e in [35]). The number of nucleosomes in the nucleus was calculated based on the genome size and nucleosome repeat length, while the numbers of nucleosomes in the individual organelles were determined based on the chromatin distribution seen in microscopy images (references in the last column). See **Methods** section for details.

Protein name	Copy number per cell	Enrichment in organelle	Reference (enrichment)	Copy number per organelle	Concentration in organelle (μM)
**Nucleolus, granular component (GC)**					
**Npm1**	22,803,667 ± 1,195,488	4.7 ± 0.3	[110] Fig. 3c (IF)	15,824,200 ± 5,502,760	60.8 ± 21.2
**Rpl23a**	9,542,514 ± 1,175,100	3.8 ± 0.1	[112] Fig. 2C (Rpl23-GFP)	6,167,027 ± 2,247,853	23.7 ± 8.7
**Rpl5**	6,506,182 ± 224,448	17.3 ± 0.9	[113] Fig. 7B (GFP-Rpl5)	5,814,283 ± 2,147,395	22.3 ± 8.3
**Ncl**	6,931,051 ± 225,884	10.1 ± 0.6	[110] Fig. 3b (IF)	5,763,236 ± 2,061,265	22.2 ± 8.0
**Lin28a**	636,709 ± 139,637	3.6 ± 0.2	[114] Fig. 1C (IF)	404,911 ± 165,255	1.6 ± 0.6
**Gnl2**	112,651 ± 21,403	∞	-	112,651 ± 21,403	0.4 ± 0.1
**Surf6**	131,920 ± 3,574	5.3 ± 0.1	[115] Fig. 1G (IF)	94,915 ± 32,763	0.4 ± 0.1
Eloa	407 ± 41	3.7 ± 0.1	[116] Fig. 5C (mCh-Eloa)	263 ± 94	0.001 ± 0.001
**Nucleolus, dense fibrillar component (DFC)**					
**Fbl**	1,863,156 ± 103,110	4.7 ± 0.2	[110] Fig. 3d (IF)	6,499 ± 189	9.8 ± 0.3
**Lin28a**	636,709 ± 139,637	3.9 ± 0.1	[114] Fig. 1C (IF)	1,918 ± 852	2.9 ± 1.3
**Polr1b**	51,889 ± 5,305	19.2 ± 1.6	[117] Fig. 1C (Polr1b-GFP)	508 ± 20	0.8 ± 0.1
Eloa	407 ± 41	5.7 ± 0.2	[116] Fig. 5C (mCh-Eloa)	2 ± 1	0.003 ± 0.002
**Transcriptional condensate (small population)**					
**Polr2a**	92,668 ± 7,370	23.1 ± 0.9	[118] Fig. 3a (IF)	6.2 ± 4.1	2.6 ± 1.7
**Brd4**	82,798 ± 11,405	10.1 ± 0.2	[43] Fig. 1A (IF)	2.6 ± 1.7	1.1 ± 0.7
**Pou5f1**	416,624 ± 1,720	1.5 ± 0.1	[119] Fig. 2a (Pou5f1-YPet)	2.0 ± 1.3	0.8 ± 0.5
**Smarca4**	227,873 ± 18,084	1.7 ± 0.1	[120] Fig. 6D (IF)	1.2 ± 0.8	0.5 ± 0.3

**Med1**	7,971 ± 1,557	49.2 ± 2.4	[43]<br>Fig. 1A (IF)	1.0 ± 0.7	0.4 ± 0.3
**Cdk9**	118,650 ± 7,659	1.5 ± 0.1	[121]<br>Movie S1 (Cdk9-mCh)	0.6 ± 0.4	0.2 ± 0.2
**Brd3**	23,528 ± 1,961	4.8 ± 0.3	[122]<br>Fig. 3C (GFP-Brd3)	0.4 ± 0.2	0.2 ± 0.1
**Ccnt1**	18,100 ± 3,762	5.2 ± 0.2	[123]<br>Fig. 4A (GFP-Ccnt1)	0.3 ± 0.2	0.1 ± 0.1
Nsd2	407 ± 41	∞	-	0.3 ± 0.3	0.1 ± 0.1
Mllt3	407 ± 41	∞	-	0.3 ± 0.3	0.1 ± 0.1
**Heterochromatin focus**					
**Trim28**	4,167,789 ± 137,584	1.5 ± 0.1	[124]<br>Fig. 5C (IF)	22,822 ± 7,114	7.9 ± 2.5
**Ube2i**	1,631,766 ± 151,670	2.9 ± 0.1	[125]<br>Fig. S2e (Ube2i-YFP)	16,035 ± 4,978	5.5 ± 1.7
**Cbx5**	1,038,731 ± 51,797	3.5 ± 0.4	[126]<br>Fig. 6D (IF)	12,190 ± 3,829	4.2 ± 1.3
**Cbx1**	522,356 ± 71,442	7.5 ± 0.8	[109]<br>Fig. 6B (IF)	11,482 ± 3,562	4.0 ± 1.2
**Hmga2**	77,331 ± 7,375	6.4 ± 0.9	[127]<br>Fig. 1A (IF)	1,513 ± 436	0.5 ± 0.2
**Mecp2**	2,614 ± 2,227	8.2 ± 0.2	[57]<br>Fig. 1 (Mecp2-GFP)	61 ± 55	0.021 ± 0.019
Kmt5c	407 ± 41	12.5 ± 2.5	[128]<br>Fig. S2C (Kmt5c-GFP)	13 ± 4	0.004 ± 0.001
Suv39h1	407 ± 41	2.6 ± 0.1	[129]<br>Fig. 3A (GFP-Suv39h1)	4 ± 1	0.001 ± 0.001
**Polycomb body**					
**Sumo3**	5,465,502 ± 801,634	1.8 ± 0.1	[130]<br>Fig. EV3A (IF)	265 ± 130	11.0 ± 5.4
**Ube2i**	1,631,766 ± 151,670	2.9 ± 0.1	[125]<br>Fig. S2e (Ube2i-YFP)	130 ± 62	5.4 ± 2.6
Cbx4	407 ± 41	∞	-	31 ± 10	1.3 ± 0.4
**Phc1**	20,041 ± 4,147	14.3 ± 0.6	[51]<br>Fig. 1d (IF)	8 ± 4	0.3 ± 0.2
Cbx2	407 ± 41	9.6 ± 0.4	[51]<br>Fig. 1d (YFP-Cbx2)	0.1 ± 0.1	0.004 ± 0.004

Table S3. Copy numbers, enrichments and resulting concentrations of candidate LLPS scaffold proteins in nucleoli, transcriptional condensates, heterochromatin foci and Polycomb bodies of mouse embryonic stem cells. Copy numbers per cell (second column) were determined based on quantitative mass spectrometry data [31], enrichment values (third column) were estimated from immunofluorescence (IF) images or from images of cells expressing a fusion of the candidate scaffold protein to a fluorescent protein (see references and annotations in the fourth column). Proteins written in italics were not detected in the respective mass spectrometry experiments and were thus assigned a value that corresponds to the estimated detection limit (407 ± 41 copies per cell). The copy numbers in large transcriptional condensates are 27-times larger than the numbers in small transcriptional condensates given here. See **Methods** section for details.

Protein name	UniProt ID	Diameter (nm) - AlphaFold	Diameter (nm) - Relaxed	Diameter (nm) - Expanded
**Nucleolus, granular component (GC)**				
**Npm1**	Q61937	7.5	9.7	12.2
**Rpl23a**	P62751	8.4	8.4	8.4
**Rpl5**	P47962	5.5	6.3	8.5
**Ncl**	P09405	9.3	16.8	21.7
**Lin28a**	Q8K3Y3	6.4	6.8	9.3
**Gnl2**	Q99LH1	8.3	13.2	24.8
**Surf6**	P70279	8.6	12.9	18.5
**Eloa**	Q8CB77	10.8	19.8	28.4
**Nucleolus, dense fibrillar component (DFC)**				
**Fbl**	P35550	5.6	8.6	10.0
**Lin28a**	Q8K3Y3	6.4	6.8	9.3
**Polr1b**	P70700	7.3	7.3	7.3
**Eloa**	Q8CB77	10.8	19.8	28.4
**Transcriptional condensate**				
**Brd4**	Q9ESU6	11.3	26.6	39.3
**Pou5f1**	P20263	7.0	11.8	17.9
**Polr2a**	P08775	9.4	20.4	41.4
**Smarca4**	Q3TKT4	11.7	21.7	42.5
**Med1**	Q925J9	10.8	26.5	41.9
**Cdk9**	Q99J95	4.7	6.3	7.6
**Brd3**	Q8K2F0	9.0	17.2	27.4
**Ccnt1**	Q9QWV9	8.9	16.9	27.4

**Nsd2**	Q8BVE8	9.6	20.1	38.7
**Mllt3**	A2AM29	10.0	16.2	22.3
**Heterochromatin focus**				
**Trim28**	Q62318	10.6	18.7	29.6
**Ube2i**	P63280	3.2	3.2	3.2
**Cbx5**	Q61686	5.3	7.4	11.0
**Cbx1**	P83917	5.8	7.2	9.9
**Hmga2**	P52927	9.3	9.3	9.6
**Mecp2**	Q9Z2D6	9.9	16.7	21.9
**Kmt5c**	Q6Q783	6.5	10.8	20.7
**Suv39h1**	O54864	5.5	5.5	5.5
**Polycomb body**				
**Sumo3**	Q9Z172	3.7	3.7	3.7
**Ube2i**	P63280	3.2	3.2	3.2
**Cbx4**	O55187	9.7	17.2	23.5
**Phc1**	Q64028	11.2	23.9	32.8
**Cbx2**	P30658	9.9	16.6	22.8

Table S4. Sizes of candidate LLPS scaffold proteins in nucleoli, transcriptional condensates, heterochromatin foci and Polycomb bodies. The predicted sizes in the third column were taken from AlphaFold [60], the sizes for more expanded conformations in the other columns are based on the scaling relationships determined in [97]. See **Methods** section for details.
