# Supplementary material for: A molecular census to elucidate the demixing mechanism of membraneless organelles
Source: Genome Biol. 2025 Oct 9;26:347. doi: 10.1186/s13059-025-03806-0 (PMC12509355; doi:10.1186/s13059-025-03806-0)
Supplement: Supplementary file 2 — Additional file 2. Web-based interactive molecular census for nucleolar GC. [file 13059_2025_3806_MOESM2_ESM.html]

Nucleolar GC


**Molecular census: Nucleolar GC**

---

|  |  |  |  |  |  |
| --- | --- | --- | --- | --- | --- |
| Nuclear volume (μm3): |  | 1320 |  | Score P: |  |
| Number of GCs: |  | 1 |  | Score P/R: |  |
| Volume of one GC (μm3): |  | 432 |  | Score P/R/N: |  |
| Total volume of all GCs combined (μm3): | | 432 |  | Prediction: |  |

---

    

| Name | UniProt | Molecules/cell | Size\_AF (nm) | Size\_rel (nm) | Size\_ext (nm) | Fraction in (all) MLOs | Molecules/MLOs | Enrichment in MLOs |
| --- | --- | --- | --- | --- | --- | --- | --- | --- |
| Npm1 | Q61937 | 22,803,667 | 7.5 | 9.7 | 12.2 |  | 15,824,200 | 4.7 |
| Rpl23a | P62751 | 9,542,514 | 8.4 | 8.4 | 8.4 |  | 6,167,027 | 3.8 |
| Rpl5 | P47962 | 6,506,182 | 5.5 | 6.3 | 8.5 |  | 5,814,283 | 17.3 |
| Ncl | P09405 | 6,931,051 | 9.3 | 16.8 | 21.7 |  | 5,763,236 | 10.1 |
| Lin28a | Q8K3Y3 | 636,709 | 6.4 | 6.8 | 9.3 |  | 404,911 | 3.6 |
| Gnl2 | Q99LH1 | 112,651 | 8.3 | 13.2 | 24.8 |  | 112,651 | Infinity |
| Surf6 | P70279 | 131,920 | 8.6 | 12.9 | 18.5 |  | 94,915 | 5.3 |
| Eloa | Q8CB77 | 407 | 10.8 | 19.8 | 28.4 |  | 263 | 3.8 |
|  |  |
| RNA (14,000 nt units) |  | 379,538 | 26.5 | 119.8 | 213.2 |  | 195,986 | 2.2 |
| Nucleosomes |  | 29,348,434 | 11.0 | 11.0 | 11.0 |  | 2,711,831 | 0.2 |
  |  |
