# Supplementary material for: A molecular census to elucidate the demixing mechanism of membraneless organelles
Source: Genome Biol. 2025 Oct 9;26:347. doi: 10.1186/s13059-025-03806-0 (PMC12509355; doi:10.1186/s13059-025-03806-0)
Supplement: Supplementary file 3 — Additional file 3. Web-based interactive molecular census for nucleolar DFC. [file 13059_2025_3806_MOESM3_ESM.html]

Nucleolar DFC


**Molecular census: Nucleolar DFC**

---

|  |  |  |  |  |  |
| --- | --- | --- | --- | --- | --- |
| Nuclear volume (μm3): |  | 1320 |  | Score P: |  |
| Number of DFCs: |  | 43 |  | Score P/R: |  |
| Volume of one DFC (μm3): |  | 1.1 |  | Score P/R/N: |  |
| Total volume of all DFCs combined (μm3): | | 47 |  | Prediction: |  |

---

    

| Name | UniProt | Molecules/cell | Size\_AF (nm) | Size\_rel (nm) | Size\_ext (nm) | Fraction in (all) MLOs | Molecules/MLOs | Enrichment in MLOs |
| --- | --- | --- | --- | --- | --- | --- | --- | --- |
| Fbl | P35550 | 1,863,156 | 5.6 | 8.6 | 10.0 |  | 279,457 | 4.7 |
| Lin28a | Q8K3Y3 | 636,709 | 6.4 | 6.8 | 9.3 |  | 82,474 | 4.0 |
| Polr1b | P70700 | 51,889 | 7.3 | 7.3 | 7.3 |  | 21,844 | 19.6 |
| Eloa | Q8CB77 | 407 | 10.8 | 19.8 | 28.4 |  | 86 | 7.2 |
|  |  |
| RNA (14,000 nt units) |  | 379,538 | 26.5 | 119.8 | 213.2 |  | 21,758 | 1.6 |
| Nucleosomes |  | 29,348,434 | 11.0 | 11.0 | 11.0 |  | 301,301 | 0.3 |
  |  |
