# Supplementary material for: A molecular census to elucidate the demixing mechanism of membraneless organelles
Source: Genome Biol. 2025 Oct 9;26:347. doi: 10.1186/s13059-025-03806-0 (PMC12509355; doi:10.1186/s13059-025-03806-0)
Supplement: Supplementary file 4 — Additional file 4. Web-based interactive molecular census for transcriptional condensates. [file 13059_2025_3806_MOESM4_ESM.html]

Transcriptional condensate


**Molecular census: Transcriptional condensates**

---

|  |  |  |  |  |  |
| --- | --- | --- | --- | --- | --- |
| Nuclear volume (μm3): |  | 1320 |  | Score P: |  |
| Number of small transcriptional condensates: |  | 969 |  | Score P/R: |  |
| Volume of one small transcriptional condensate (μm3): |  | 0.004 |  | Score P/R/N: |  |
| Number of large transcriptional condensates: |  | 14 |  | Prediction: |  |
| Volume of one large transcriptional condensate (μm3): |  | 0.1 |
| Total volume of all transcriptional condensates combined (μm3): | | 5.3 |

---

    

| Name | UniProt | Molecules/cell | Size\_AF (nm) | Size\_rel (nm) | Size\_ext (nm) | Fraction in (all) MLOs | Molecules/MLOs | Enrichment in MLOs |
| --- | --- | --- | --- | --- | --- | --- | --- | --- |
| Polr2a | P08775 | 92,668 | 9.4 | 20.4 | 41.4 |  | 7,894 | 23.1 |
| Brd4 | Q9ESU6 | 82,798 | 11.3 | 26.6 | 39.3 |  | 3,225 | 10.1 |
| Pou5f1 | P20263 | 416,624 | 7.0 | 11.8 | 17.9 |  | 2,493 | 1.5 |
| Smarca4 | Q3TKT4 | 227,873 | 11.7 | 21.7 | 42.5 |  | 1,544 | 1.7 |
| Med1 | Q925J9 | 7,971 | 10.8 | 26.5 | 41.9 |  | 1,314 | 49.2 |
| Cdk9 | Q99J95 | 118,650 | 4.7 | 6.3 | 7.6 |  | 710 | 1.5 |
| Brd3 | Q8K2F0 | 23,528 | 9.0 | 17.2 | 27.4 |  | 445 | 4.8 |
| Nsd2 | Q8BVE8 | 407 | 9.6 | 20.1 | 38.7 |  | 407 | Infinity |
| Mllt3 | A2AM29 | 407 | 10.0 | 16.2 | 22.3 |  | 407 | Infinity |
| Ccnt1 | Q9QWV9 | 18,100 | 8.9 | 16.9 | 27.4 |  | 370 | 5.2 |
|  |  |
| RNA (2,790 nt units) |  | 1,904,491 | 15.5 | 44.2 | 72.9 |  | 10,776 | 1.4 |
| Nucleosomes |  | 29,348,434 | 11.0 | 11.0 | 11.0 |  | 176,429 | 1.5 |
  |  |
