# Supplementary material for: A molecular census to elucidate the demixing mechanism of membraneless organelles
Source: Genome Biol. 2025 Oct 9;26:347. doi: 10.1186/s13059-025-03806-0 (PMC12509355; doi:10.1186/s13059-025-03806-0)
Supplement: Supplementary file 5 — Additional file 5. Web-based interactive molecular census for heterochromatin foci. [file 13059_2025_3806_MOESM5_ESM.html]

Heterochromatin focus


**Molecular census: Heterochromatin foci**

---

|  |  |  |  |  |  |
| --- | --- | --- | --- | --- | --- |
| Nuclear volume (μm3): |  | 1320 |  | Score P: |  |
| Number of foci: |  | 10 |  | Score P/R: |  |
| Volume of one focus (μm3): |  | 4.8 |  | Score P/R/N: |  |
| Total volume of all foci combined (μm3): | | 48 |  | Prediction: |  |

---

    

| Name | UniProt | Molecules/cell | Size\_AF (nm) | Size\_rel (nm) | Size\_ext (nm) | Fraction in (all) MLOs | Molecules/MLOs | Enrichment in MLOs |
| --- | --- | --- | --- | --- | --- | --- | --- | --- |
| Trim28 (Kap1) | Q62318 | 4,167,789 | 10.6 | 18.7 | 29.6 |  | 228,220 | 1.5 |
| Ube2i (Ubc9) | P63280 | 1,631,766 | 3.2 | 3.2 | 3.2 |  | 160,350 | 2.9 |
| Cbx5 (HP1α) | Q61686 | 1,038,731 | 5.3 | 7.4 | 11.0 |  | 120,840 | 3.5 |
| Cbx1 (HP1β) | P83917 | 522,356 | 5.8 | 7.2 | 9.9 |  | 114,820 | 7.5 |
| Hmga2 | P52927 | 77,331 | 9.3 | 9.3 | 9.6 |  | 15,130 | 6.4 |
| Mecp2 | Q9Z2D6 | 2,614 | 9.9 | 16.7 | 21.9 |  | 610 | 8.1 |
| Kmt5c (Suv4-20h2) | Q6Q783 | 407 | 6.5 | 10.8 | 20.7 |  | 130 | 12.4 |
| Suv39h1 (Kmt1a) | O54864 | 407 | 5.5 | 5.5 | 5.5 |  | 40 | 2.9 |
|  |  |
| RNA (2,790 nt units) |  | 1,904,491 | 15.5 | 44.2 | 72.9 |  | 60,279 | 0.9 |
| Nucleosomes |  | 29,348,434 | 11.0 | 11.0 | 11.0 |  | 1,761,859 | 1.7 |
  |  |
