# Supplementary material for: A molecular census to elucidate the demixing mechanism of membraneless organelles
Source: Genome Biol. 2025 Oct 9;26:347. doi: 10.1186/s13059-025-03806-0 (PMC12509355; doi:10.1186/s13059-025-03806-0)
Supplement: Supplementary file 6 — Additional file 6. Web-based interactive molecular census for Polycomb foci. [file 13059_2025_3806_MOESM6_ESM.html]

Polycomb body


**Molecular census: Polycomb bodies**

---

|  |  |  |  |  |  |
| --- | --- | --- | --- | --- | --- |
| Nuclear volume (μm3): |  | 1320 |  | Score P: |  |
| Number of bodies: |  | 13 |  | Score P/R: |  |
| Volume of one body (μm3): |  | 0.04 |  | Score P/R/N: |  |
| Total volume of all bodies combined (μm3): | | 0.52 |  | Prediction: |  |

---

    

| Name | UniProt | Molecules/cell | Size\_AF (nm) | Size\_rel (nm) | Size\_ext (nm) | Fraction in (all) MLOs | Molecules/MLOs | Enrichment in MLOs |
| --- | --- | --- | --- | --- | --- | --- | --- | --- |
| Sumo3 | Q9Z172 | 5,465,502 | 3.7 | 3.7 | 3.7 |  | 3,445 | 1.6 |
| Ube2i (Ubc9) | P63280 | 1,631,766 | 3.2 | 3.2 | 3.2 |  | 1,690 | 2.6 |
| Cbx4 | O55187 | 407 | 9.7 | 17.2 | 23.5 |  | 407 | Infinity |
| Phc1 | Q64028 | 20,041 | 11.2 | 23.9 | 32.8 |  | 104 | 13.2 |
| Cbx2 | P30658 | 407 | 9.9 | 16.6 | 22.8 |  | 1 | 8 |
|  |  |
| RNA (2,790 nt units) |  | 1,904,491 | 15.5 | 44.2 | 72.9 |  | 455 | 0.6 |
| Nucleosomes |  | 29,348,434 | 11.0 | 11.0 | 11.0 |  | 17,810 | 1.5 |
  |  |
